# A multicentre, double-blind, randomised, controlled, parallel-group study of the effectiveness of a pharmacist-acquired medication history in an emergency department

**DOI:** 10.1186/1472-6963-13-337

**Published:** 2013-08-29

**Authors:** Jesus Becerra-Camargo, Fernando Martinez-Martinez, Emilio Garcia-Jimenez

**Affiliations:** 1 Pharmacy Department, Universidad Nacional de Colombia, Ciudad Universitaria, Edificio 450 oficina 204, Bogotá 14490, Colombia; 2 Pharmacy Department, Universidad de Granada, Granada University’s pharmaceutical care research institute, Paseo Cartuja, S/N, 18071 Granada, Spain

## Abstract

**Background:**

Admission to an emergency department (ED) is a key vulnerable moment when patients are at increased risk of medication discrepancies and medication histories are an effective way of ensuring that fewer errors are made. This study measured whether a pharmacist-acquired medication history in an ED focusing on a patient’s current home medication regimen, and available to be used by a doctor when consulting in the ED, would reduce the number of patients having at least 1 medication discrepancy related to home medication.

**Methods:**

This multicentre, double-blind, randomised, controlled parallel-group study was conducted at 3 large teaching hospitals. Two hundred and seventy participants were randomly allocated to an intervention (n = 134) or a standard care (n = 136) arm. All consecutive patients >18 years old admitted through the ED were eligible. The intervention consisted of pharmacists conducting a standardised comprehensive medication history interview focusing on a patient’s current home medication regimen, prior to being seen by a doctor. Data recorded on the admission medication order form was available to be used by a doctor during consultation in the ED. The admission medication order form was given to doctors at a later stage in the control arm for them to amend prescriptions. The effect of the intervention was assessed primarily by comparing the number of patients having at least 1 admission medication discrepancy regarding medication being taken at home. Secondary outcomes concerned the characteristics and clinical severity of such medication discrepancies.

**Results:**

The intervention reduced discrepancies occurring by 33% (p < 0.0001; 0.1055 odds ratio, 0.05-0.24 95% confidence interval), despite recall bias. Regarding total discrepancies, omitting medication occurred most frequently (55.1%) and most discrepancies (42.7%) were judged to have the potential to cause moderate discomfort or clinical deterioration.

**Conclusions:**

A pharmacist-acquired medication history in an ED focusing on a patient’s current home medication regimen available to be used by a doctor at the time of consulting in the ED reduced the number of patients having at least 1 home medication-related discrepancy.

**Trial registration:**

Current Controlled Trials ISRCTN63455839.

## Background

Medication histories are an effective way of contributing to a decrease in hospital admission-related medication discrepancies [1]. An accurate history of medication use is an important part of patient appraisal on admission [2]. The completeness of such medication use history depends on different factors concerning a particular patient, including the time available for interview, language barriers, illness severity, cognitive status and a patient’s familiarity with his or her medication regimen [3]. A pharmacist’s involvement as part of the care-team in an admission clinic can improve patient safety [4]. Studies have shown that a pharmacist-acquired medication history is more comprehensive than that obtained by other health professionals [5-7], as pharmacists represent an ideal resource because they possess the pertinent knowledge and training [7-12].

Unintentional medication discrepancies frequently occur on admission to an emergency department (ED); up to 60% of patients admitted to an in-patient unit have at least 1 unintended medication discrepancy regarding their home medication regimen and the admission orders [2,3,12]. The most common error lies in omitting a medication being taken at home [11,12]; this problem concerns the multidisciplinary health-care team’s difficulty in accessing a complete and accurate home medication list. Hospital admission is thus an interface in the care process regarding a potential gap in the transfer of medication-related information [4].

The Joint Commission on Accreditation of Healthcare Organizations has defined medication reconciliation (MedRec) as the process of comparing a patient’s medication orders to all of the medications that such patient has been taking. An accurate and up-to-date medication history is considered an essential component of safe and effective medical practice. MedRec is aimed at avoiding medication errors such as omissions, duplications, dosing errors or drug interactions. It should be made during every care stage/transition where new medications are ordered or existing orders may be rewritten. Transitions in care include changes in setting, service, practitioner or care level [13].

It has been hypothesised that a hospital involving the pharmacist as part of a multidisciplinary team in the ED for supporting medication being prescribed on admission and incorporating MedRec will prevent and reduce home medication-related discrepancy frequency on admission.

### Aim

This study’s primary objective was to evaluate whether a pharmacist-acquired medication history in an ED focusing on a patient’s current home medication regimen, and available to be used by doctor at the time of consulting in an ED, would reduce the number of patients having at least 1 medication discrepancy related to home medication.

## Methods

### Trial design

A multicentre, double-blind, randomised and controlled parallel-group trial study was conducted in accordance with the provisions of the Declaration of Helsinki (1996) and Good Clinical Practice (GCP) guidelines. All participants gave their written informed consent. The trial was registered as Current Controlled Trial ISRCTN63455839. The allocation ratio for the intended numbers of participants in each comparison group was 1:1. The report involved using consolidated standards of reporting trials (CONSORT) [14].

### Testing the survey questionnaire

The procedure for validating data collection forms and standardising the admission medication order form involved 30 patients (ten participants per hospital). Particular emphasis was placed on examining whether the researchers were able to complete the questionnaires in full without difficulty (i.e. responding to the patients’ answers) and the length of time required for doing so. This was verified using a simulated exercise.

The whole team received formal training on how to complete a MedRec form, including a description of data-collection tools and procedures. Two pharmacists per hospital independently filled out the forms, different percentages of agreement being obtained between hospitals (100% Fundacion Cardio Infantil, 60% Hospital Samaritana and 50% at Hospital San Carlos). Differences and the team were evaluated, agreement being reached by consensus.

### Participants

The study was conducted from October 26^th^ to November 30^th^ 2012 at 3 large teaching hospitals in Bogota, Colombia. All consecutive patients (18 years or older) who had been admitted to an ED, taking at least one medication or had been prescribed a minimum of one prescription medication before admission, who had been assessed as triage I and II on admission and who had been hospitalised for at least 24 hours were eligible for inclusion. Patients were classified as triage I or II (triage I meant patients having ventilator, haemodynamic and neurological stability, suffering a condition representing a potential threat to life or loss of limb or organ if they were not to have received prompt medical intervention; triage II referred to when patients had a condition which might have progressed to becoming serious and were judged as requiring emergency assessment and were likely to require inpatient treatment) [15,16]. Patients were enrolled on weekdays, weekends and holidays (i.e. 24 hours per day). Patients were excluded if they had been scheduled for discharge on the same day, were not able to answer the questions needed to complete the study, were unable to communicate due to language difficulties, were under psychiatric care, had a medical record of dementia or confusion and/or were unable to give their consent.

### Pharmacist intervention

The intervention consisted of a pharmacist-acquired medication history in an ED focusing on a patient’s current home medication regimen documented on the admission medication order form (F1) which was available to be used by a doctor when consulting in an ED. Doctors verified the data with patients and indicated which home medications were to be reordered, suspended or discontinued. This resulted in an accurate and comprehensive history of a patient’s current home medication regimen, called medication order form verified with patient (F1V).

### On admission to an ED

One team of research pharmacists employed for this study conducted a standardised, comprehensive medication history interview, focusing on current home medication regimen for all patients included in the study, prior to being seen by a doctor. A thorough history of all regular medication use (prescription, non-prescription, nutritional supplements, vitamins, over-the-counter, herbal, vaccines, drugs and diagnostic contrast or radioactive agents, parenteral nutrition, blood products and intravenous solutions) was ascertained using all the following sources of information: patient and/or caregiver interview, a check of the last prescription and an inspection of the medicines carried by a patients (i.e. in the ED). Pharmacists conducted telephone interviews with caregivers or family members when patients were unable to clarify their medication regimen. Relevant demographic and medical data was collected and documented on F1.

F1 was used by a doctor during consultation for issuing prescriptions in an inpatient ED (just for the intervention group). By checking boxes, the doctor verified with a patient and indicated which home medications were to be reordered, suspended or discontinued (F1V). For patients who were in the control arm, F1 was given to the doctors at a later stage for them to amend prescriptions.

### Follow-up medication

Another team of regular pharmacists blinded to intervention status in the hospitals being studied reviewed each medical chart regarding all the drugs prescribed 24 hours after having been admitted to an ED. The data came from various information sources including a patient’s computerised hospital medical record, F1, the physician-recorded medication history, the nurse-recorded medication history, interviews with patients, medication administration records and demographic information. The pharmacist also attempted to verify with the patient if any medication changes had been made since their clinical assessment (i.e. on admission to an ED). This was documented in the list of medications prescribed by a doctor during 24 hours in an ED (F2).

### Medicine reconciliation

MedRec was used for comparing a patient’s current home medications to medications prescribed 24 hours after having been admitted to an ED to see whether patients’ home medications were also prescribed by a doctor in an ED. This was done by an independent team (pharmacist and doctor blinded to intervention status) and took place the day after admission.

**Figure 1:**
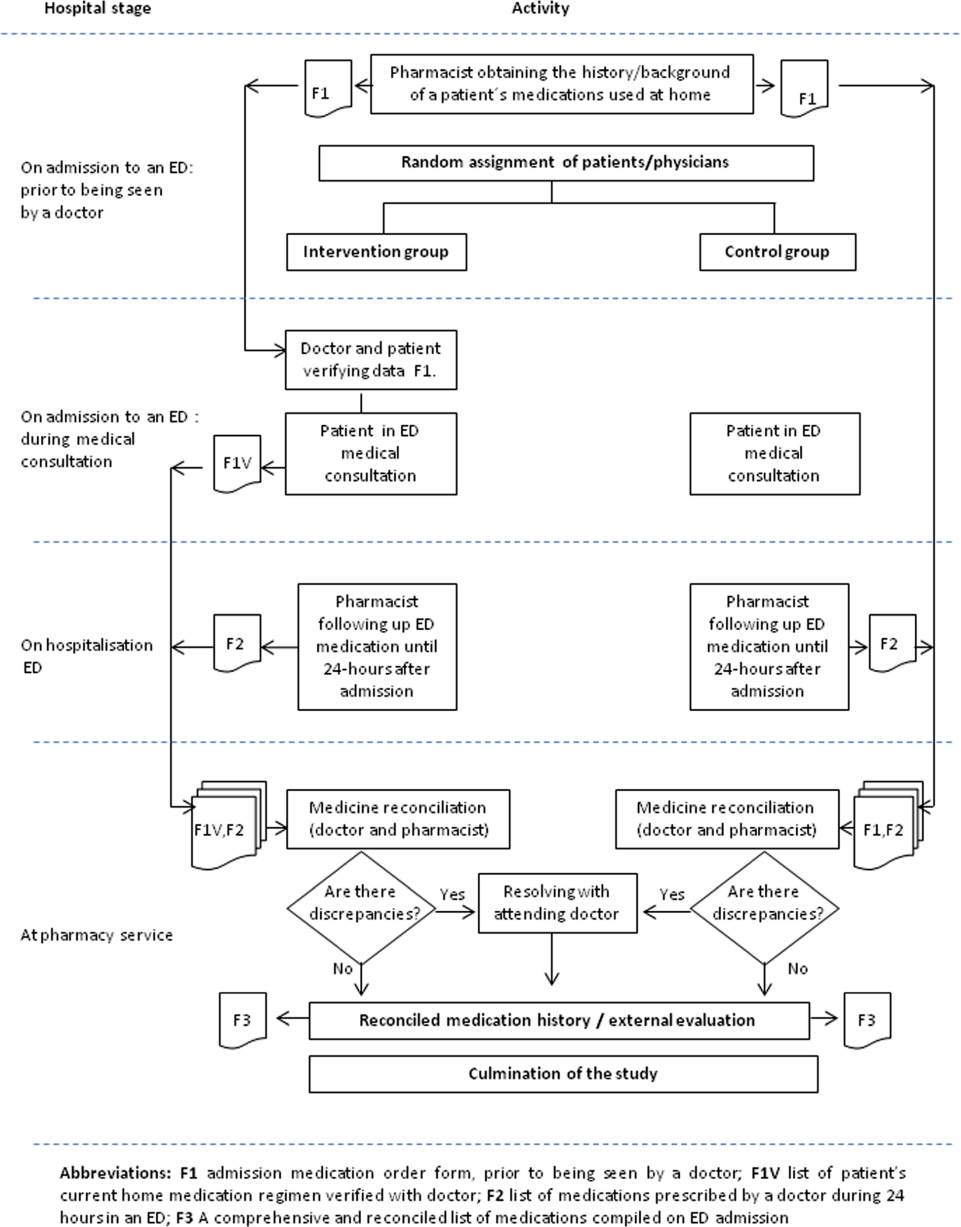
Study design.

If incongruity was detected and the reason had not been documented in the medical record, this was clarified with the medical team and patient. External evaluation was made by the chief of each hospital’s ED after MedRec had ended; this person then resolved any discrepancies with each doctor. Following MedRec, further medication required doctors to write separate medication orders (see Figure 1).

### Standard group

Patients in the control group received standard care; this included doctors documenting medication histories in admission notes and nurses reviewing medication orders for appropriateness. Doctors wrote in-patient orders during consultation without having access to F1. The medication information was entered on each medical chart forming part of a hospital’s electronic health records. Pharmacists were not routinely involved in documenting patients’ admission medication histories; this function is primarily the admitting resident doctor or medical student’s responsibility at the institutions involved in the present study.

### Outcomes

The effect of the intervention was assessed by comparing the number of patients having at least 1 admission medication discrepancy regarding medication being taken at home. Secondary outcomes concerned the characteristics and clinical severity of such medication discrepancies. Admission discrepancies were defined as any medication clarification related to current home medication made whilst being cared for in an ED. They could have been associated with any of the following: drug, dosage, frequency, administration route, appropriateness of restarting medication, therapeutic duplicity and/or medications lacking indication. Discrepancies were identified using a systematic approach (i.e. MedRec).

The clinical severity of medication discrepancies was independently assessed by two clinical pharmacists blinded to the patient data collection forms; they classified each type of medication discrepancy according to its potential to cause harm, the degree of effect being adapted from the method used by Cornish et al. [2]. Disagreements were resolved by discussion and consensus was reached for all discrepancies. The degree of effect for each medication discrepancy was defined as follows. Class 1 discrepancies were classified as being those unlikely to cause patient discomfort or clinical deterioration, Class 2 those having the potential to cause moderate discomfort or clinical deterioration and Class 3 discrepancies had the potential to result in severe discomfort or clinical deterioration.

Allergies were identified using part of F1; this only focused on requiring information about food and drug allergies and type of allergic reaction.

Interactions were identified using the Drug Interaction Checker; it also displayed any interactions between the drugs being chosen and food and explained the mechanism for each drug interaction (interaction significance level could have been major, moderate or minor) [17].

### Sample size

A multicentre study was conducted. The baseline number of patients having at least 1 medication discrepancy was 30%; this was used for calculating sample size. A 2-tailed Chi square test (α = 0.05 and ß = 0.20) gave a minimum enrolment of 120 patients in each arm for the study to have sufficient power, assuming that the intervention arm would reduce the number of patients having at least 1 of admission medication discrepancy related to home medication by 50%. This was increased to 135 patients in each group to compensate for dropouts. The reference values used for establishing sample size were taken from previous studies [4].

### Randomising

Patients and doctors were randomly assigned to the intervention or standard care arm using random-number computer generation in Microsoft Excel. Allocation by each randomisation manager was daily and depended on the number of patients, doctors and residents per shift. Randomisation involved 3 blocks of 90 numbers, one per each hospital. The allocation group could have equated odd and even numbers for interventions A and B, respectively, to ensure equal allocation.

### Allocation concealment mechanism

The combined coded numbers concerning intervention allocation were concealed in sequentially-numbered, sealed, opaque envelopes and kept by the clinical trials group at the Universidad Nacional de Colombia (UNALCO, Bogota). The assignments were also concealed in sequentially-numbered containers, according to the allocation sequence. It was ensured that all envelopes were numbered in advance and that they were equal in weight and similar in appearance. It was guaranteed that the envelopes were opened sequentially and only after a participant’s name and other details had been written on the assignation list. Stringent procedures were used for ensuring enrolment before randomisation. Two exactly similar copies of the randomisation list were prepared; one was used by the randomisation manager and the other copy was kept under lock and key by the emergency coordinator. The envelope was made of cardboard to render it impermeable to intense light. An audit trail was created.

### Implementation

Each block of 90 numbers was sent from the central office to a person who acted as the randomisation manager in each hospital. A nurse (epidemiologist) not involved in caring for the trial patients and independent of the site investigator was responsible for trial allocation and record-keeping (i.e. the randomisation manager). The randomisation schedule was thus concealed from all care providers, ward doctors and other research personnel. The pharmacists worked in different shifts to obtain the records. The MedRec procedure was adapted and standardised from the Institute for Healthcare Improvement “toolkit” that provides extensive details on where and how to reconcile medications, how to implement procedures and provides sample flow-charts, algorithms and forms [18,19].

### Blinding

No patient, nurse, doctor, investigator or any other medical or nursing staff in an ED was aware of the intervention assignments for the duration of the study; neither the patients nor the doctors could distinguish between the intervention and control group. All patients were interviewed by pharmacists. They were supplied with uniforms and ID cards similar to those of the hospital workers; they were involved in admission work.

The doctors were assigned to receive only patients in the intervention or control group during their shifts to ensure blinding. The randomisation manager placed an F1 into the medical chart so that a doctor would think that it was regular documentation. If they asked about the form, the randomisation manager told them it concerned an on-going pilot study. The randomisation manager was instructed to report any suspected breach of the masking procedures.

The forms used were made to look the same as the forms used in the hospitals, the logo, colours and fonts being exactly alike so it seemed that the doctor was filling in just another new form. All statistical analysis involved maintaining the masking. Analysis was completed before the randomisation code was broken at the end of the completed trial. Each researcher sent the data online via an information system link provided by the statistics office. All records were checked.

### Statistical methods

An exact *X*^2^ test was used to investigate differences in the percentage of patients having at least 1 medication discrepancy between treatment groups; baseline characteristics were compared using the exact *X*^2^ test as appropriate. Multivariate logistic regression analysis was used to investigate predictors of at least 1 medication discrepancy.

Comparisons were made between the intervention and control groups by logistic regression analysis for binary responses using odds ratios (Cox proportional hazards model). Linear regression analysis was used for continuous responses using differences and Poisson regression analysis for incidences; fractions were used for comparisons. All tests were 2-tailed and a test result was deemed statistically significant at p < 0.05. All statistical analysis involved using R statistical software.

## Results

### Participant flow

**Figure 2:**
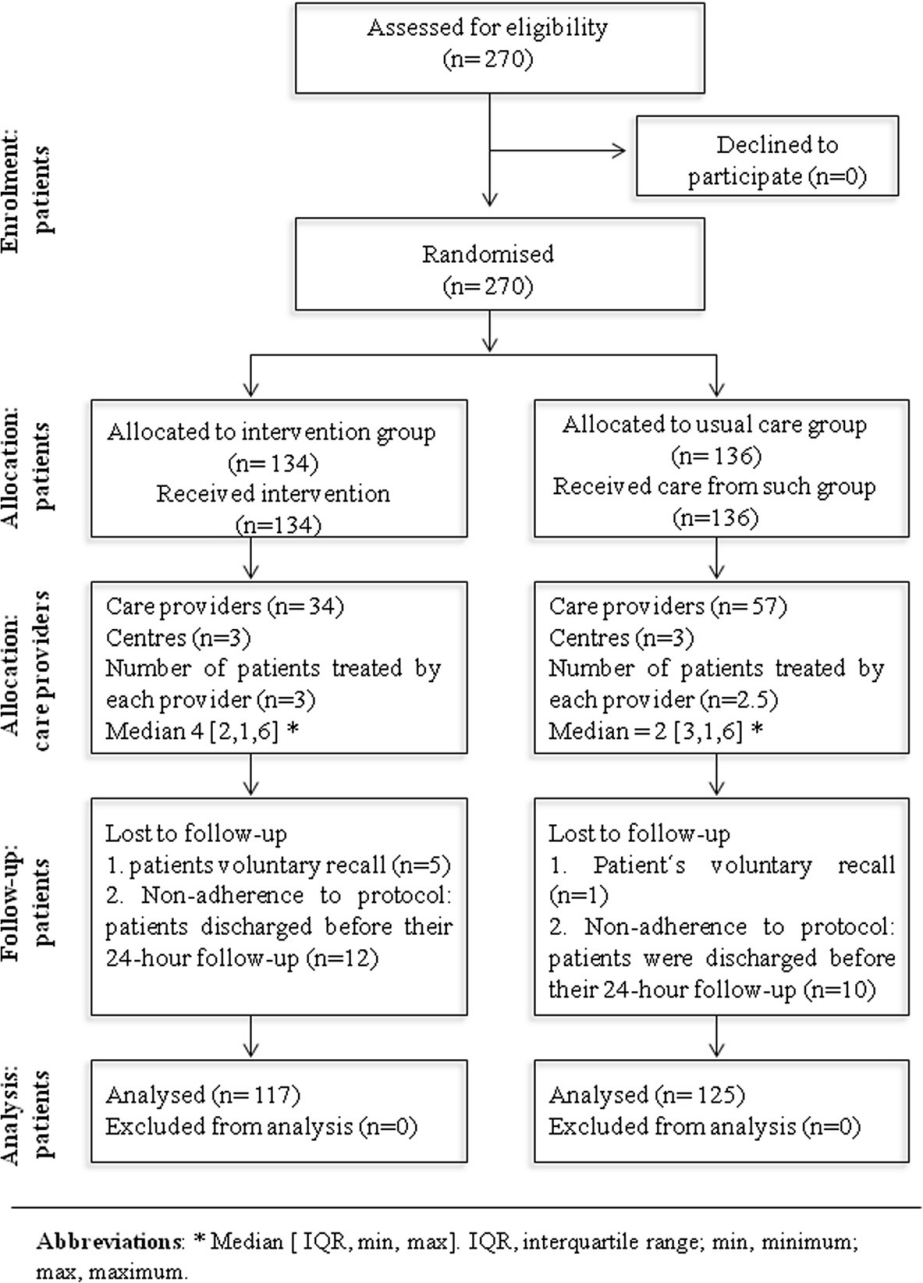
Participant flowchart.

The 270 patients who were randomised and selected by consecutive sampling for the study (134 intervention and 136 controls) were cared for by each of the 3 randomised teams and by 91 admitting doctors. Twenty-two patients (12 interventions and 10 controls) had been assessed and ranked incorrectly during triage and were discharged on the same day. They were excluded from the main study analysis and the most common reason for patient exclusion was non-adherence to protocol because they had been discharged before their 24-hour follow-up. Six patients (5 interventions and 1 control) voluntarily decided to leave the hospital and seek care at another hospital and they were consequently excluded. Figure 2 shows the flow of participants throughout the trial.

### Baseline data

Table 1 give patients’ baseline demographic and clinical characteristics regarding the intervention and standard care arms. There were no statistically significant differences between both treatment arms. Patient characteristics in the 2 study arms were similar.

**Table 1:** The study population’s baseline demographic and clinical characteristics

**Characteristics**	**All groups**	**Control**	**Intervention**	**Value**	**p–value**
	**242 (100)**	**125 (51.65)**	**117 (48.35)**		
**Age, mean ± SD**	59 ± 19	58 ± 20	59 ± 18	−0.5355^a^	0.5928
**Gender**				0.2233^b^	0.6365
Female	140 (57.9)	70 (56.0)	70 (59.8)		
Male	102 (42.1)	55 (44.0)	47 (40.2)		
**Educational level**				0.0572^b^	0.9964
No schooling	19 (7.9)	10 (8.0)	9 (7.7)		
Elementary school	105 (43.4)	54 (43.2)	51 (43.6)		
High school	81 (33.5)	43 (34.4)	38 (32.5)		
Undergraduate	33 (13.6)	17 (13.6)	16 (13.7)		
No information	4 (1.7)	1 (0.8)	3 (2.5)		
Social status*				6.7379^b^	0.08074
Strata 1 (lowest level)	135 (82.3)	59 (76.6)	76 (87.4)		
Strata 2 (low - medium)	22 (13.4)	12 (15.6)	10 (11.5)		
Strata 3 (medium)	2 (1.2)	1 (1.3)	1 (1.1)		
Strata 6 (highest level)	5 (3.1)	5 (6.5)	0 (0.0)		
**Teaching hospitals**				5.1145^b^	0.07752
Fundacion Cardio Infantil	82 (33.9)	36 (28.8)	46 (39.3)		
San Carlos	78 (32.2)	48 (38.4)	30 (25.6)		
La Samaritana	82 (33.9)	41 (32.8)	41 (35.1)		
**No. of hospitalisations, median (IQR, min, max)**	0 (1, 0, 12)	0 (1, 0, 10)	0 (1, 0, 12)	−0.2168^a^	0.8285
**No. of co-morbidities, median (IQR, min, max)**	1 (2, 0, 4)	1 (2, 0, 4)	1 (1, 0, 4)	0.4219 ^a^	0.6735
**Type of comorbidity**				11.6316^b^	0.392
Arthritis/osteoporosis	16 (5.5)	11 (7.2)	5 (3.6)		
Malignant diseases	12 (4.1)	4 (2.6)	8 (5.7)		
Cardiovascular disorder	90 (30.8)	47 (30.7)	43 (31.0)		
Diabetes mellitus	22 (7.5)	12 (7.9)	10 (7.2)		
Epilepsy/Parkinson’s disease	10 (3.4)	7 (4.6)	3 (2.2)		
Pulmonary disease	18 (6.2)	13 (8.5)	5 (3.6)		
Gastrointestinal disease	15 (5.2)	7 (4.6)	8 (5.8)		
Hypertension	14 (4.8)	6 (3.9)	8 (5.8)		
Hypothyroidism	22 (7.5)	8 (5.2)	14 (10.0)		
Metabolism disease	18 (6.2)	8 (5.2)	10 (7.2)		
Urinary infection	15 (5.1)	7 (4.6)	8 (5.7)		
Other	40 (13.7)	23 (15.0)	17 (12.2)		
**No. of medicines, median (IQR, min, max)**	4 (4, 1, 12)	4 (4, 1, 16)	4 (4, 1, 14)	−0.3299^a^	0.7418
**Medicine group**				17.208^b^	0.3066
Anti-infective for systemic use	39 (3.7)	15 (2.8)	24 (4.6)		
Anti-inflammatory drugs	60 (5.6)	32 (5.9)	28 (5.4)		
Cardiovascular system	252 (23.6)	130 (23.9)	122 (23.3)		
Alimentary tract and metabolism	194 (18.2)	102 (18.8)	92 (17.6)		
Systemic hormonal preparations	31 (2.9)	12 (2.2)	19 (3.6)		
Respiratory system	65 (6.1)	33 (6.1)	32 (6.1)		
Blood and blood-forming organs	93 (8.7)	51 (9.4)	42 (8.0)		
Nervous system	168 (15.7)	90 (16.5)	78 (14.9)		
Vitamins and nutrients	39 (3.7)	21 (3.7)	18 (3.4)		
Herbal medicine	21 (2.0)	7 (1.3)	14 (2.7)		
Over-the-counter medicine	75 (7.0)	37 (6.8)	38 (7.3)		
Other	30 (2.8)	14 (2.6)	16 (3.1)		
**Allergic reaction**				0^b^	1
No	194 (80.2)	100 (80.0)	94 (80.3)		
Yes	48 (19.8)	25 (20.0)	23 (19.7)		
**No. of interactions**					
Minor^c^, median (IQR, min, max)	1 (0, 1, 3)	1 (0, 1, 3)	1 (0, 1, 2)	−0.3943	0.6942
Moderate^d^, median (IQR, min, max)	1 (1, 1, 7)	1 (1, 1, 7)	1 (1, 1, 3)	0.9524	0.3416
Major^e^, median (IQR, min, max)	1 (0, 1, 4)	1 (0, 1, 2)	1 (0, 1, 4)	−1.4242	0.1787

*Abbreviations*: ^a^t student; ^b^Chi-square; *SD* (standard deviation). *IQR,* interquartile range; min, minimum; max, maximum. ^*^ Social status is not important data during the interview on admission to the ED at the San Carlos teaching hospital.

^c^**Minor**: Minimal clinical significance, involving minimising risk, assessing risk and considering an alternative drug, taking steps to circumvent interaction risk and/or instituting a monitoring plan.

^d^**Moderate**: Moderate clinical significance, usually avoiding combinations, being used only in special circumstances.

^e^**Major**: High clinical significance, avoiding combinations; the risk of the interaction would outweigh the benefits.

### Effect of the intervention

For the primary end point, 117 (93.6%) of the 125 patients had at least 1 admission medication discrepancy in the standard care arm, compared to 71 (60.7%) of 117 patients in the intervention care arm (p < 0.0001; 0.1055 odds ratio (OR), 0.05-0.24 95% confidence interval (CI)). The overall discrepancy rate was 3.35 per patient (SD 3.32); it was 4.23 (SD 3.26) in the standard care arm and 2.43 (SD 3.14) in the intervention arm.

Doctor/pharmacist agreement on identifying allergies was also studied using Cohen’s association coefficient (*K* = 0.434; 95%CI; p < 0.001), showing a moderate level of agreement between both types of reviewer as the pharmacists identified 48 patients suffering allergies and the doctors 23.

The association between discrepancies and baseline patient characteristics was also evaluated (Table 2). Increasing age was a weak predictor of having a medication discrepancy only in the univariate model (1.02 OR, 1.01-1.04 95%CI). Regarding the study population’s clinical characteristics in the univariate model, the number of medications taken at home (1.45 OR, 1.25-1.72 95%CI; p = 5.51E-06) and number of co-morbidities (1.56 OR, 1.14-2.19 95%CI; p = 0.00734) increased the risk of discrepancy most whilst healthcare-service settings regarding ED admission and intervention significantly decreased such risk.

**Table 2:** Association between patients’ baseline characteristics and medication discrepancies

**Characteristics**	**Univariate logistic regression**		**Multivariate logistic regression**	
	**Odds ratio (95%CI)**	**p-value**	**Odds ratio (95%CI)**	**p-value**
**Age**	1.02 (1.01 - 1.04)	0.00496	1.02 (0.99 - 1.04)	0.2475
**Being female**	1.37 (0.74 - 2.52)	0.312	1.27 (0.56 - 2.87)	0.5659
**Educational level**				
No schooling	0.83 (0.20 - 3.69)	0.800466	1.21 (0.15 - 10.33)	0.8579
Elementary level	0.77 (0.26 - 1.98)	0.603804	0.80 (0.16 - 3.79)	0.7851
High school level	0.70 (0.23 - 1.85)	0.492108	0.92 (0.21 - 3.69)	0.9116
**Emergency department (Teaching hospitals)**				
San Carlos	0.43 (0.18 - 0.98)	0.0496	0.26 (0.07 - 0.89)	0.0366
La Samaritana	0.32 (0.14 - 0.70)	0.00548	0.28 (0.07 - 1.00)	0.0553
**Number of hospitalisations**	1.18 (0.93 - 1.69)	0.242	1.00 (0.74 - 1.48)	0.9825
**Allergic reaction**	1.55 (0.71 - 3.79)	0.297	0.74 (0.24 - 2.34)	0.602
**Number of comorbidities**	1.56 (1.14 - 2.19)	0.00734	0.89 (0.53 - 1.55)	0.6774
**Number of home medications**	1.45 (1.25 - 1.72)	5.51E-06	1.62 (1.31 - 2.06)	3.12E-05
**Intervention**	0.11 (0.04 - 0.23)	4.65E-08	0.04 (0.01 - 0.11)	1.16E-09

*Abbreviations*: *CI*, confidence interval.

Patients’ clinical conditions accounted for the decrease in discrepancies revealed by multivariate analysis. Due to the association between variables, the number of medicines being taken increased discrepancy risk (1.62 OD, 1.31-2.06 95%CI; p = 3.12E-05) while the healthcare-setting regarding ED admission decreased discrepancy risk (0.26 OR, 0.07-0.89 95%CI; p = 0.0366). Pharmacist intervention was also associated with an increased effect concerning reducing discrepancy risk (0.04 OR, 0.01-0.11 95%CI; p = 1.16E-09). The effect of such intervention remained statistically significant (p < 001) after adjustment for all other predictor variables.

### Medication discrepancy characteristics and clinical severity

The most common discrepancy concerned the omission of home medication being reordered (55.1%); 66.3% of omission discrepancies were in the standard arm and 34.3% in the intervention arm. This was followed by incorrect or omitted dose and being slow to restart drug therapy. Over-the-counter-medicines were omitted in 100% of the cases. Other types of medication discrepancies are summarised in Table 3. The two most common classes involved in discrepancy were cardiovascular agents (23.6%) and alimentary tract and metabolism agents (17.0%).

**Table 3:** Characteristics regarding medication discrepancies

**Type of medication discrepancy**	**All groups**	**Standard care arm**	**Intervention arm**	**Value**	**p-value**
	**242 (100)**	**125 (51.65)**	**117 (48.35)**		
**Type of discrepancy**				97.5313	< 2.2e-16
Incorrect or omitted dose	165 (20.4)	83 (15.7)	82 (29.0)		
Therapeutic duplication	5 (0.6)	4 (0.8)	1 (0.3)		
Incorrect or omitted frequency	6 (0.7)	6 (1.1)	0 (0.0)		
No indication	2 (0.3)	2 (0.4)	0 (0.0)		
Drug omission	447 (55.1)	350 (66.3)	97 (34.3)		
Too soon to restart drug therapy	26 (3.2)	15 (2.8)	11 (3.9)		
Slow to restart drug therapy	156 (19.2)	64 (12.1)	92 (32.5)		
Inappropriate route	4 (0.5)	4 (0.8)	0 (0.0)		
Number of discrepancies, median (IQR, min, max)	3 (4, 0, 15)	3 (4, 0, 15)	1 (4, 0, 13)	4.3812	1.765e-05

*Abbreviations*: *IQR*, interquartile range; in, minimum; max, maximum.

A higher percentage of patients who were interviewed by pharmacists were identified as using home medication (457, 82.8%) compared to those in the standard care arm (255, 42.5%) regarding 1,169 medications consumed by patients in the study.

The likelihood that a medication discrepancy might have caused discomfort and/or clinical deterioration was appraised and categorised. Fleiss’ kappa coefficient was used (κ = 0.829; p = 0) [20]. There was very good agreement among evaluators in judging the potential clinical effect of medication discrepancy.

Most discrepancies (42.7%) were judged to have the potential to cause moderate discomfort or clinical deterioration; 33.4% of the discrepancies were deemed unlikely to cause harm and 23.9% were judged to have the potential to cause severe discomfort or clinical deterioration (Table 4).

**Table 4:** Discrepancy type and potential severity

**Type of discrepancy**	**No.**	**Class 1**^**a**^	**Class 2**^**b**^	**Class 3**^**c**^
Incorrect or omitted dose	165	39 (23.6)	32 (19.4)	94 (57.0)
Therapeutic duplication	5	4 (80.0)	0 (0.00)	1 (20.0)
Incorrect or omitted frequency	6	0 (0.00)	4 (66.7)	2 (33.3)
Slow to restart drug therapy	156	28 (18.0)	103 (66.0)	25 (16.0)
No indication	2	0 (0.00)	2 (100)	0 (0.00)
Drug omission	447	190 (43.0)	187 (42.0)	70 (15.0)
Too soon to restart drug therapy	26	10 (38.0)	14 (54.0)	2 (8.0)
Inappropriate or omitted route	4	0 (0.00)	4 (100)	0 (0.00)
**Total**	811	271 (33.4)	346 (42.7)	194 (23.9)

^a^Class 1: discrepancies unlikely to cause patient discomfort or clinical deterioration.

^b^Class 2 discrepancies which could cause moderate discomfort or clinical deterioration.

^c^Class 3 discrepancies potentially resulting in severe discomfort or clinical deterioration.

Four hundred and sixty-three interactions were identified: 8.7% major, 68.2% moderate and 23.1% minor. The major interactions were associated with the inflammatory, blood and central and peripheral nervous systems. One hundred and fourteen patients had at least one interaction, 63.2% in the standard care arm and 36.8% in the intervention arm. Intervention reduced the occurrence of interactions.

### Assessing the process

All discrepancies were communicated to the chief of each hospital’s ED after data collection had ended; this person then resolved discrepancies with each doctor. Our results confirmed that pharmacists’ interventions were well-received by ED doctors, having a 96% acceptance rate. The result was unknown in only 4% of the suggested actions.

The median time required for each pharmacist to complete the medication record for each patient was 29.5 minutes (IQR = 22.25 minutes, minimum 6 minutes, maximum 135 minutes).

## Discussion

Several publications [5,9,21,22] have demonstrated that pharmacist-acquired medication histories in an ED have led to reducing discrepancies; our results were consistent with previous studies. The effect of the intervention reduced discrepancies occurring by 33% (p < 0.0001; 0.1055 OR, 0.05-0.24 95%CI), despite patient recall bias.

Although every attempt was made to interview patients or family members and inspect prescription vials or medication bottles for all study subjects, medications could not be inspected in many cases; the interviewer/researcher relied on written medication lists provided by the patient, caregiver or family or calling his/her house in such situations.

In spite of the intervention, 71 patients (60.7%) in the intervention arm still had at least 1 ED admission medication discrepancy related to home medication. Emphasis was placed on patients reporting the use of at least 1 medication during ED admission. Error rates may have differed regarding services other than admission or among patients taking more than 1 medication. Our results were consistent with previous studies [21,22] which have reported that medication discrepancies when being admitted to an ED are frequent and clinically significant. Omission regarding over-the-counter medications in our study was 100%.

Obtaining medication history can be challenging. One study has reported that unintentional medication discrepancies were more often due to errors in recording medication history than errors reconciling the history with medication orders. It also reported that relying on family members or caregivers as sources of medication information represented a risk factor [23].

Our study incorporated MedRec when being admitted to an ED. It has proved useful for improving patient safety by reducing medication discrepancies before harm can occur to patients [8]. The reduction of medication discrepancies in the intervention arm reflected the importance of obtaining accurate and complete medication histories.

Some studies [5,9,24] have reported that pharmacists are ideal for supporting a multidisciplinary healthcare team because of their increased familiarity with medications. Our results agreed with previous studies which reported that pharmacists involved in ED admission could increase medication detection level and identify more medications per patient than doctors or nurses when noting their medication history [7,24], i.e. such histories were more accurate and complete.

Pharmacists used multiple sources for gathering a complete medication history for proactively issuing medication orders to support doctors making prescriptions during ED admission. Dawson et al.*,*[25] have shown that doctors obtained an average of 79% of the complete drug history regarding prescription drug use and 45% for over-the-counter drug use, whereas pharmacists obtained a 100% complete history for both categories of drug use. Todd et al.*,*[26] has stated that pharmacists documented significantly more medication doses and dosage schedules than physicians did (614 cf 446 and 614 cf 404, respectively) (p ≤ 0.001 for both comparisons) and 614 medications were identified by pharmacists for the 55 patients interviewed, compared to 556 identified by doctors (p ≤ 0.001).

In some earlier non-randomised studies of pharmacist-conducted medication histories and assessment before admission to an ED [21], the most common type of discrepancy involved the omission of a medication which a patient was taking at home (57%); our findings confirmed such report (55.1%).

Hayes et al., [27] found that allergy documentation was recorded for 62 patients in a control group (79%) compared to 60 patients in the study group (100%) (p = 0.001). Twenty-five patients were identified in our study suffering from allergies in the intervention arm whilst the standard care arm identified 23 (p < 0.001). Pharmacists identified an almost equal number of allergies as did the doctors.

Overall, a reduction in discrepancies was due to intervention, expected clinical conditions (number of medications being taken) and a particular ED setting. Although the hospitals participating in the study were selected as they had very similar general characteristics, it was revealed that an ED healthcare setting significantly affected the risk of discrepancies occurring.

The hospitals may have had different results because staff training/procedures were different and training admitting physicians and medical students could have had some impact. Significant barriers often occur regarding accurate and complete medication information being obtained during admission related to patients who go to hospitals due to the differing demographics of the populations which they serve. The amount and availability of resources for healthcare attention and the hospitals’ capacity for attending patients could have influenced the results. These observations were made during data collection.

Evaluating the potential clinical impact of the unintentional discrepancies identified during our study showed that 23.9% were judged to have had the potential to cause a patient severe discomfort. Potential harm was driven by the type of discrepancy (incorrect or omitted dose). An example would be a patient who was admitted with cardiac arrhythmia; 150 mg propafenone was ordered by the ED doctor (R2) because it had been noted during the first patient interview (R1) that he had been taking propafenone; however, the ED doctor did not know that a lower dose had already been ordered by a cardiologist 6 weeks earlier (i.e. based on later scrutiny of the patient’s record by MedRec staff – R3).

Zed et al., [28] evaluated incidence, severity and preventability of medication-related visits to an ED, establishing that severity had been classified as moderate; of all patients having medication discrepancies at the time of ED admission in our study, more than two thirds of such discrepancies had the potential to cause moderate harm.

The number of patients having at least one interaction was very high (36.8%), compared to studies elsewhere [29]. This could have been related to different definitions or prescribing practice. Further analysis is required for identifying what might have motivated the above differences (our group is currently working on an adverse-events-based paper to extend this line of research). Interactions do not always represent contraindications for use, but knowledge and an appreciation of them before prescription is essential for the safe use of medication. This should thus be considered when a doctor decides to continue with a set prescription after evaluating any possible interaction.

It was estimated that a pharmacist would have to spend 29.5 minutes per patient to provide this service for patients in a similar ED. However, such calculation was based on uninterrupted work-flow times and may have underestimated the actual time required outside a research setting. A consultation with just a pharmacist in an ED focusing on a medication list may be a solution. Models maximising the use of a pharmacist should be evaluated in future research.

This study had several limitations. Despite the study being conducted in teaching hospitals, the results may not be generalised to other settings because an ED setting was a factor regarding the risk of a discrepancy appearing. Future research could examine the effect of ED admission setting and blocking; a cluster study should thus be carried out.

It is acknowledged that the validity of any method for scoring medication discrepancy is difficult to assess because there is currently no gold standard for comparison. A reconciled history (F3) should provide the gold standard for identifying home medication use; however, this could have been a limitation of this study. Patient or caregiver reports were relied on, in conjunction with collateral information from medication vials whenever possible.

Error rates may differ regarding services other than an ED concerning admissions which are elective or involve a transfer from another healthcare facility, or concerning patients taking more than 1 medication. Our findings may not have been representative of other institutions using processes different to MedRec for admission. Eligible patients were not followed-up beyond the study; thus, we are not aware of the effect of such ED admission process on medical outcomes.

The rating method used for assessing the potential severity of the discrepancies and F1 questionnaire used during a medication history interview has not been validated. Inter-rater agreement was also not evaluated as interviewing the same patient twice could have led to recall bias.

The hospitals involved in this study are developing a MedRec pathway which will incorporate some strategies based on the present study’s findings. The next phase will involve an assessment of medication discrepancies once the new MedRec protocol is in place. The data presented herein has suggested that recording medication histories on admission could be improved. Future research could involve a cost-benefit analysis of the intervention for implementing such initiative.

## Conclusions

To improve patient care and minimise unintended discrepancies in an ED, the healthcare system should explore ways for involving a pharmacist as part of the multidisciplinary team to improve ED admission medication history accuracy thereby improving patient safety during admission. A pharmacist-acquired medication history in an ED focusing on a patient’s current home medication regimen available to be used by a doctor at the time of consulting in an ED reduced the number of patients having at least 1 medication discrepancy related to home medication.

## Ethics approval

The protocol and supporting documents were reviewed, approved and registered by Ethics Committees for Clinical Research: Fundacion Cardio Infantil (DDI-376 September 18^th^, 2012, Dr. José Sinay Arévalo Leal, MD, PhD. President), San Carlos teaching hospital (FHS C-OCC 100–12 13^th^ August 2012, Dr. Juan Pablo Robayo MD, secretary) and Samaritana teaching hospital (142 June 27^th^, 2012, Dr. Omar Velandia MD, secretary).

## Competing interests

The authors declare no competing interests regarding the content of this article.

## Authors’ contributions

BJ, submitting author, directed data abstraction, analysis and interpretation. MF, involved in design, devised the initial research proposal concept. MF and GJE guided the team of pharmacist researchers for this article covering all sections. BJ drafted our results and table section. MF and GJE drafted the discussion and conclusion sections. MF provided mentorship for our research team and acquired funding. All authors have read and approved the final manuscript.

## Pre-publication history

The pre-publication history for this paper can be accessed here:

http://www.biomedcentral.com/1472-6963/13/337/prepub
